# A Comprehensive Review of Thrombocytopenia With a Spotlight on Intensive Care Patients

**DOI:** 10.7759/cureus.27718

**Published:** 2022-08-05

**Authors:** Ratnam K Santoshi, Reema Patel, Neil S Patel, Varinder Bansro, Gurdeep Chhabra

**Affiliations:** 1 Internal Medicine, Critical Care Medicine, and Anesthesia, Springfield Clinic, Springfield, USA; 2 Medicine, Lake Erie College of Osteopathic Medicine, Erie, USA; 3 Physiology and Neurobiology, University of Maryland, College Park, USA; 4 Internal Medicine, University of Maryland Capital Region Health, Largo, USA; 5 Hematology and Medical Oncology, University of Maryland Capital Region Health, Largo, USA

**Keywords:** transfusion practices, thromboelastography (teg), sepsis induced thrombocytopenia, pregnancy-induced thrombocytopenia, thrombotic thrombocytopenia syndrome, thrombocytopenia, immune-mediated thrombocytopenia, post-operative thrombocytopenia, drug-induced thrombocytopenia, thrombotic thrombocytopenic thrombocytopenia

## Abstract

Thrombocytopenia is a common entity seen in ICU patients and is associated with increased morbidity such as bleeding and transfusions, and mortality in ICU patients. Various mechanisms such as decreased platelet production, sequestration, destruction, consumption, and sometimes a combination of these factors contribute to thrombocytopenia. An understanding of the mechanism is essential to diagnose the cause of thrombocytopenia and to help provide appropriate management. The management strategies are aimed at treating the underlying disorder, such as platelet transfusion to treat complications like bleeding. Several studies have aimed to provide the threshold for platelet transfusions in various clinical settings and recommend a conservative approach in the appropriate scenario. In this review, we discuss various pathophysiological mechanisms of thrombocytopenia and the diverse scenarios of thrombocytopenia encountered in the ICU setting to shed light on the varied thresholds for platelet transfusion, alternative agents to platelet transfusion, and future directions for the implementation of thromboelastography (TEG) in multiple clinical scenarios to assist in the administration of appropriate blood products to correct coagulopathy.

## Introduction and background

Thrombocytopenia is a condition in which platelet counts are < 150x10^9^/L. It can be further characterized as mild (100-149x10^9^/L), moderate (59-99x10^9^/L) and severe (<50x10^9^/L). It is a hematologic pathology that is commonly found in ICU patients with a prevalence of 8.3% to 67.6% and an incidence of 14% to 44% [1]. This wide range is due to variability in age, the severity of illness, and presentation such as medical and surgical etiologies. The significant contributing factors to the development of thrombocytopenia in ICU are the severity of illness, organ dysfunction, sepsis, shock, and renal failure [1]. Thrombocytopenic patients are at an increased risk of bleeding requiring transfusion of various blood products, increased length of stay, and increased risk of mortality in patients admitted to the ICU [1]. 

Studies have shown that complications and mortality rates are significantly increased in critical care patients if platelet levels take longer than four days to recover. Persistent thrombocytopenia at or over two weeks is associated with higher mortality than platelet levels that reach baseline levels by day four [2]. Akca et al. described the time course of platelet count in critically ill patients and showed that the mortality rate goes up to 66% in ICU patients if thrombocytopenia persists for 14 days after initial ICU admission, and is reduced to 16% if the platelet levels are successfully restored [3]. The recovery rate can also serve as an important indicator for prognosis, as survivors had an average of a 30 × 10^9^/L x day increase, and non-survivors had a ≤ 6 × 10^9^/L x day increase [2].

The various pathophysiological mechanisms involved in thrombocytopenia are decreased production, increased destruction of platelets by immune or non-immune mechanisms, and increased sequestration in the reticuloendothelial system [1]. Understanding the mechanism involved in thrombocytopenia and the various differential diagnoses is key to appropriate management. A highly structured diagnostic approach enables early detection of common causes and allows for rational use of platelet transfusions [2]. There are various transfusion triggers to transfuse platelets depending on the patient’s current ongoing bleeding conditions, risk of bleeding, preparation for surgical procedures, as well as prophylactic transfusions in certain specific conditions. To this day, there are no unified guidelines regarding these thresholds and it varies on an institutional basis and is at the clinician's discretion. 

Platelet physiology

Platelet activation is the first step in hemostasis and occurs in a three-step process, namely adhesion, activation, and aggregation. Platelets adhere to the exposed subendothelial surface of injured vessel walls which are mediated by the von Willebrand factor (vWF). Platelets then change shape and release adenosine diphosphate and triphosphate, serotonin, fibrinogen, vWF, and factor V. Clopidogrel irreversibly binds to the P2Y12 adenosine diphosphate receptor, blocking platelet activation and aggregation. During shape change, glycoprotein IIb/IIIa receptors are exposed which bind fibrinogen and initiate reversible platelet aggregation. The intravenous antiplatelet agents such as abciximab, eptifibatide, and tirofiban inhibit fibrinogen binding during this stage. In the final stage, arachidonic acid is released and converted to thromboxane A2, promoting irreversible platelet aggregation and resulting in a platelet plug. Aspirin acts by irreversibly acetylating thromboxane A2. Finally, platelets provide the phospholipid membrane necessary to complete the coagulation cascade [4]. 

Pathophysiological mechanisms involved in thrombocytopenia

The major mechanisms causing low platelet count are pseudothrombocytopenia, hemodilution, decreased platelet production, increased platelet consumption, increased sequestration of platelet, and platelet destruction. The most common causes of thrombocytopenia in ICU are drug-induced secondary to heparin, glycoprotein IIb/IIIa (GP2b3a) inhibitors alcohol, antibiotics, sepsis, major bleeding, microangiopathic hemolytic anemias such as thrombocytopenic thrombotic purpura (TTP), hemolytic uremic syndrome (HUS), and disseminated intravascular coagulation (DIC). Table 1 describes the various pathophysiological mechanisms and etiology of thrombocytopenia [5]. Understanding the different mechanisms and the etiology is crucial to managing thrombocytopenia appropriately in intensive care units. For instance, low platelets caused by major bleeding are managed by transfusing platelets, whereas in conditions such as TTP and HUS, platelet transfusion is contraindicated and plasmapheresis is the treatment of choice. Heparin-induced thrombocytopenia and thrombosis (HITT) require timely diagnosis, immediate cessation of heparinoids, and initiation of alternative anticoagulants such as direct thrombin inhibitors. Decreased production of platelets can occur from underlying bone marrow suppression from cytotoxic agents or alcohol, aplastic anemia, infections, malignancies, and nutritional deficiencies. Physiologically, platelets survive in the circulation for seven to 10 days with up to 33% sequestered in the spleen. Splenic sequestration secondary to cirrhosis and portal hypertension can lead to a reduction in post-transfusion platelet augmentation. The most common etiology in intensive care unit patients is a combination of increased destruction and consumption such as in sepsis and DIC. Thrombocytopenia in sepsis can be explained by various mechanisms such as complement activation; increased markers of coagulation activation such as thrombin release; hemophagocytosis; a disintegrin and metalloproteinase with a thrombospondin type 1 motif, member 13 (ADAMTS13) depletion; and release of histones. 

**Table 1:** Pathophysiological mechanisms and etiology of thrombocytopenia SLE: Systemic lupus erythematosus, HIV: Human immunodeficiency virus, EBV: Epstein-Barr virus, CMV: Cytomegalovirus, DIC: Disseminated intravascular coagulation, ECMO: Extracorporeal membrane oxygenation, IABP: Intra-aortic balloon pump, HCV: Hepatitis C virus, CLL: Chronic lymphocytic leukemia, HELLP: Hemolysis elevated liver enzymes and low platelet, TTP: Thrombotic thrombocytopenic purpura, HUS: Hemolytic uremic syndrome

Pathophysiological mechanisms and differential diagnosis of thrombocytopenia	Clinical scenario
Pseudothrombocytopenia	If the sample is clotted, collect the sample in an anticoagulant such as citrate GPIIbIIIa inhibitors
Hemodilution	Infusion of fluids and or plasma; massive transfusion in cases of major bleeding
Decreased platelet production	Aplastic anemia; alcohol or drugs; viral infections: HIV, HCV, EBV, CMV; hematologic malignancies: leukemia, lymphoma, myelodysplasia metastatic malignancies; Nutritional deficiency: vitamin B12, drug-induced folate deficiency; radiation and chemotherapy
Increased platelet consumption	Major blood loss; trauma; DIC; sepsis extracorporeal circuits: post-cardiopulmonary bypass, IABP, renal dialysis, ECMO
Increased platelet sequestration	Liver cirrhosis, osteomyelofibrosis, hypersplenism: portal hypertension, congestive heart failure, hematologic malignancies, lipid storage disorders
Platelet destruction	Immune thrombocytopenia: SLE, HCV, CLL; drug-induced: quinine, quinidine, trimethoprim/sulfamethoxazole, vancomycin, penicillin, rifampin, carbamazepine, ceftriaxone, ibuprofen, mirtazapine, oxaliplatin, suramin, heparin, Gp2a/3b inhibitors, ADP receptor antagonists; microangiopathic hemolytic anemia: TTP, HUS, HELLP, DIC; post-transfusion purpura; passive alloimmune thrombocytopenia

Diagnostic methodology for thrombocytopenia

A careful and thorough history and physical exam is the linchpin. A history of past medical history, comorbidities, medications, recent viral illness, travel history, and current presentation to the hospital will aid in the diagnosis of the cause of thrombocytopenia. Existing thrombocytopenia may denote an undiagnosed myelodysplastic syndrome (MDS) or lymphoproliferative disorder which may also lead to decreased production of other cell lines. In the case of chronic immune thrombocytopenic purpura (ITP), other cell lines will be normal. In cases of cirrhosis leading to portal hypertension and splenic sequestration, there might be thrombocytopenia associated with leukopenia. A history of alcoholism will support the diagnosis of bone marrow failure as a cause of pancytopenia. A complete blood count for the concomitant decrease in other cell lines, peripheral smear to identify specific etiologies and coagulation studies help in identifying the specific causes of thrombocytopenia. In critical care patients, thrombocytopenia is most often caused by sepsis, DIC, drugs, or malnutrition. Bone marrow aspirations may be performed in certain cases where the cause is unknown and/or the blood samples show abnormalities in other cell lines [6]. 

Physical exam findings of current bleeding, petechiae, gastrointestinal bleeding, splenomegaly, lymphadenopathy, and fundal hemorrhage should be looked for. Peripheral smear adds to the diagnosis such as giant platelets in pseudothrombocytopenia, schistocytes and spherocytes in TTP, left shift and bandemia in sepsis, blasts in leukemia, large platelets suggestive of peripheral destruction, macrocytosis, target cells in liver disease, alcohol use, nutritional deficiency (vitamin B12, folate) and tear-drop cells in MDS. 

## Review

Pseudothrombocytopenia

A factitiously low platelet count can occur due to clumping and underestimation of platelet count by automated particle counter leading to pseudothrombocytopenia. Clumping of platelets occurs due to in vitro agglutination by ethylenediaminetetraacetic acid (EDTA)-dependent IgG or IgM antibodies. Pseudothrombocytopenia due to EDTA-dependent antibodies can be confirmed by repeating the platelet count with another anticoagulant such as citrate, heparin, or oxalate. These antibodies can persist transiently or indefinitely and are not associated with any abnormalities related to bleeding or thrombosis. An EDTA-independent pseudothrombocytopenia can occur secondary to autoantibodies such as cold agglutinins. Giant platelets and platelet satellitism can also lead to spuriously low platelets on automated platelet counts. Thrombocytopenia on automated blood count should be confirmed by peripheral smear examination before any further workup or treatment is undertaken [7].

The use of GP2b3a inhibitors such as abciximab, eptifibatide, and tirofiban can give pseudothrombocytopenia with both EDTA and citrate. Ruling out pseudothrombocytopenia is required before establishing the diagnosis of real thrombocytopenia caused by GP2b3a inhibitors and before further actions to stop these agents are attempted. The use of GP2b3a inhibitors in patients requiring percutaneous intervention (PCI) is very crucial to prevent in-stent thrombosis. These agents are also associated with thrombocytopenia and patients developing thrombocytopenia are at increased risk of worse outcomes such as death, myocardial infarction, cardiac bypass surgery, or additional PCI than patients without thrombocytopenia. Once pseudothrombocytopenia has been ruled out and true thrombocytopenia secondary to these agents is established, stopping these agents and changing to direct thrombin inhibitors (DTI) such as bivalirudin or lepirudin is recommended [8]. Platelet transfusion is advised for life-threatening bleeding complications or if platelet counts <10,000/μL. Percutaneous intervention patients often are on heparin infusions along with GP2b3a inhibitors. Heparin-induced thrombocytopenia (HIT) should be ruled out as well with an antiplatelet factor 4 antibodies and serotonin release assay. Unlike GP2b3a inhibitors that are associated with bleeding complications, HIT is associated with thrombosis. Heparin-induced thrombocytopenia is managed with DTI agents, and platelet transfusions should be avoided [8]. 

Immune thrombocytopenic purpura (ITP) 

Immune thrombocytopenic purpura is an isolated condition of low platelets usually <100,000/microL and a generalized purpura rash but normal white blood cells and hemoglobin. It was previously known as idiopathic thrombocytopenic purpura. However, since it is due to IgG-mediated autoantibodies against platelet membrane proteins such as GP2b3a complex, GP Ib/IIa, and GP VI, it is known as immune thrombocytopenic purpura. The binding of the antibodies to the platelet results in accelerated clearance of these platelets by the spleen. There is usually an inciting event such as infection or immune alteration that sets off the cascade that leads to platelet destruction (Table 2) [9]. 

**Table 2:** Causes of immune thrombocytopenic purpura HIV: Human immunodeficiency virus

Infection	Immune alteration
Viral (most common) such as HIV, hepatitis C, cytomegalovirus, varicella-zoster virus, or bacterial	Antiphospholipid syndrome, systemic lupus erythematosus, Evans syndrome, hematopoietic cell transplantation, chronic lymphocytic leukemia, common variable immunodeficiency, and autoimmune lymphoproliferative syndrome

Evans syndrome is ITP associated with autoimmune hemolytic anemia. Immune thrombocytopenic purpura is a diagnosis of exclusion and patients usually present with a history of mucocutaneous bleeding, sudden development of a petechial rash, or bruising [10]. Children with ITP and platelet count less than 30,000/ μL, should be restricted from contact/collision sports and any activities associated with increased bleeding risk from trauma. According to the American Society of Hematology 2019 guidelines, the management of ITP is based on age and severity of thrombocytopenia. Treatment consists of corticosteroids, intravenous immunoglobulin (IVIG) or anti-D immunoglobulin, rituximab, danazol, cyclophosphamide, azathioprine, or splenectomy (Table 3) [9]. 

**Table 3:** Management of immune thrombocytopenic purpura (ITP)

Severity of ITP	Management
Asymptomatic or mild mucosal bleeding with platelet count >30,000/μL	Observation
Mild mucosal bleeding with Platelet count <30,000/μL	Steroids for <6 weeks
Platelet count <20,000/μL	Admit to hospital and steroids for <6 weeks
Chronic ITP (steroid dependent or unresponsive for over three months)	Thrombopoietin receptor agonists (i.e., eltrombopag or romiplostim or avatrombopag), rituximab, and splenectomy
Third-line agents	Fostamatinib (spleen tyrosine kinase inhibitor), danazol, azathioprine, cyclosporine, mycophenolate

Drug-induced thrombocytopenia

In order to attribute thrombocytopenia due to medication use, several criteria must be met, namely thrombocytopenia should occur following drug administration, discontinuation of medication will enable the patient to recover and maintain that recovery, all other causes of thrombocytopenia have been excluded, and if the medication is readministered it will most likely result in recurrence of thrombocytopenia [11]. Drug-induced thrombocytopenia can be immune-mediated destruction or due to underlying factors such as bone marrow suppression from various etiologies. A systematic evaluation conducted by Arnold et al. determined that there are 153 drugs that are known to have an increased risk of inducing thrombocytopenia [11]. Of those, quinine, quinidine, trimethoprim/sulfamethoxazole, vancomycin, penicillin, rifampin, carbamazepine, ceftriaxone, ibuprofen, mirtazapine, oxaliplatin, and suramin; the GP2b3a inhibitors abciximab, tirofiban, eptifibatide, and heparin are considered to be definite causes [11]. The most common mechanism and drugs contributing to DIT are listed in Table 4 [10]. 

**Table 4:** Drug-induced thrombocytopenia and their mechanism of action

Mechanism of action	Drugs
Decreased production	Chemotherapeutic agents: busulfan, cyclophosphamide, daunorubicin, methotrexate, 6 mercaptopurine, vinca alkaloids; estrogens; ethanol; linezolid; thiazide diuretics
Immune-mediated destruction	Abciximab, amphotericin B, aspirin, carbamazepine, chloroquine, cimetidine, ranitidine, clopidogrel, digoxin, eptifibatide, heparin, meropenem, phenytoin, piperacillin, quinine, bactrim, valproic acid, vancomycin
Unknown mechanisms	Fluconazole, ganciclovir, nitrofurantoin, rifampin, valganciclovir

Heparin-induced thrombocytopenia

The decrease in platelet count after the initiation of heparin in the first few days could be associated with type 1 HIT. It is non-immune mediated, reversible, and resolves spontaneously with a platelet nadir above 100x109/L. It is not associated with any complications [10]. As for type 2 HIT, it is an immune-mediated > 50% drop in platelet count occurring five to 10 days after exposure to heparin for the first time, or a more rapid decrease in platelet count can occur with prior exposure to heparin products in the past 30 days. Type 2 HIT is caused by antibodies against platelet factor 4 (complexed to heparin). The platelet counts are usually below 100x109/L. Localized skin necrosis or erythema at the site of injection can occur and systemic symptoms such as fever, wheezing, and tachycardia from heparin boluses can occur. Continued heparin administration in patients with type 2 HIT is associated with severe thrombosis in up to 50% of patients. The associated thrombosis are deep vein thrombosis (DVT), pulmonary embolism (PE), or more unusual acute arterial occlusions, which can be life-threatening. The 4Ts score has been developed to identify patients at risk for heparin-induced thrombocytopenia (Table 5) [12]. 

**Table 5:** The 4Ts scoring system IV UFH: Intravenous unfractionated heparin

The 4 Ts	2 points	1 point	0 points
Thrombocytopenia	Platelet count decrease >50% and platelet nadir >20,000/μL	Platelet count decreases 30% to 50% or platelet nadir 10,000-19,000/μL	Platelet count decrease <30 or platelet nadir <10,000μL
Timing of platelet count decrease	Onset between days 5 to 10 or platelet count decrease ≤1 day (with heparin exposure within 30 days)	Onset between days 5 to10, onset after day 10, or decrease ≤1 day (heparin exposure 30 to 100 days ago)	Onset <4 days without recent exposure
Thrombosis or other sequelae	New thrombosis (confirmed), skin necrosis, acute systemic reaction after IV UFH bolus	Progressive or recurrent clot, non-necrotizing (e.g., erythematous) skin lesions, suspected thrombosis (not proved)	None
Other causes of thrombocytopenia	None apparent	Possible	Definite

**Figure 1:**
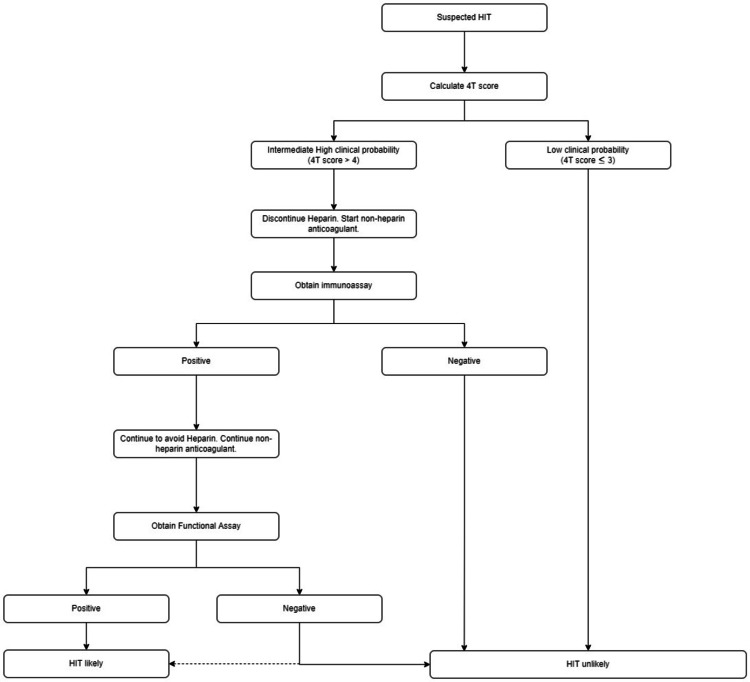
HIT diagnosis algorithm as per American Society of Hematology 2018 guidelines HIT: Heparin-induced thrombocytopenia

For high and intermediate-risk patients, it is recommended that they stop heparin and start a non-heparin anticoagulant is recommended, submit to an enzyme immunoassay (EIA) that will serve as a sensitive test, as well as a serotonin release assay (SRA) which serves as a specific test for HIT. An EIA identifies anti-platelet factor 4/heparin IgG antibody (PF4 Ab) that activates platelets triggering serotonin release which is a functional assay and is highly specific for the diagnosis of HIT. Heparin-induced thrombocytopenia is considered unlikely for low clinical probability patients on the 4Ts scoring (Figure 1) [13].

Management of HIT is primarily discontinuation of heparin immediately, avoiding heparin for life, and considering it as a medication allergy. An alternate anticoagulant should be started (unless the patient is actively bleeding). Alternate agents include intravenous argatroban, bivalirudin, danaparoid, subcutaneous fondaparinux, or a non-vitamin K antagonist oral anticoagulant such as rivaroxaban. Warfarin should not be the initial alternate anticoagulant because antithrombotic efficacy requires three to five days of therapy, during which time declining levels of protein C increase the thrombotic risk. Transitioning to warfarin is safe after thrombocytopenia resolves. Anticoagulation should be continued for two to three months in patients with HIT without documented thromboembolic events, or three to six months for patients with HIT with associated thrombosis [10]. 

Sepsis

Sepsis is defined as “life-threatening organ dysfunction caused by a dysregulated host response to infection” [14]. The majority of pathologies in intensive care patients are sepsis, acute lung injury (ALI)/adult respiratory distress syndrome (ARDS), and shock accounting for most of the morbidity and mortality in ICU patients [15]. The normal hemostasis in the body is controlled through a complex network of prothrombotic and fibrinolytic activity. Coagulopathy in sepsis is caused by inflammation-induced upregulation of coagulation cascade, dysfunctional anticoagulant, and fibrinolytic systems leading to varying degrees of dysregulated hemostasis ranging from thrombosis to DIC and bleeding. There is increased platelet activation and aggregation in sepsis which combined with tissue injury, hypoxia, enhanced host immune response and endotoxemia leads to cellular necrosis/apoptosis, microvascular dysfunction, and abnormal coagulation. Thrombocytopenia has been found to be an independent risk factor for increased mortality and morbidity in sepsis and septic shock patients and the magnitude of platelet reduction correlates to the severity of the illness. Thrombocytopenia in sepsis is multifactorial including decreased production secondary to drug-induced deficiencies, infection, bone marrow suppression, and nutritional deficiencies. In sepsis, there is also increased elimination from splenic sequestration or increased consumption such as DIC. Hemodilution, immune-mediated and spurious thrombocytopenia are also other causes of thrombocytopenia in sepsis. Acquired plasma protease ADAMTS13 deficiency has also been described in sepsis and systemic inflammatory response syndrome. The functional decrease in ADAMTS13 or incremental decline in ADAMTS13 activity in sepsis has been postulated to cause excessive platelet activation and thrombocytopenia in sepsis and ARDS [15]. 

The florid inflammatory response in sepsis activates the coagulation cascade leading to blood clots and an increased risk of thrombosis. The initial inflammatory response is beneficial to defend against the infectious process, however, when sepsis worsens the hemostasis equilibrium is disrupted leading to microvascular thrombosis and consequently leading to organ failure. It is very crucial to understand precisely when the procoagulant response to infection transforms from a favorable to destructive process. There is ongoing research in the field of anticoagulant-targeted therapies in sepsis-related coagulopathy with the aim to improve morbidity and mortality by regulating platelet response [16]. 

Thrombotic microangiopathies

Thrombotic microangiopathies are a group of disorders characterized by microangiopathic hemolytic anemia, thrombocytopenia, and microthrombi leading to ischemic tissue injury [17]. Microangiopathic hemolytic anemia is the hallmark of thrombotic microangiopathy. It is a process of red blood cell destruction within the microvasculature accompanied by thrombocytopenia due to platelet activation and consumption. Thrombotic thrombocytopenic purpura (TTP) and hemolytic uremic syndrome (HUS) are primary forms of thrombotic microangiopathies. Regardless of the etiology, thrombotic microangiopathy is a hematologic emergency that requires prompt treatment.

Thrombotic thrombocytopenic purpura is broadly defined as thrombotic microangiopathy occurring in the context of severe ADAMTS13 deficiency (< 10%). The ADAMTS13 is a normal enzyme in plasma that cleaves large forms of the coagulation protein vWF into smaller functional subunits. The congenital form is Upshaw Schulman syndrome and the acquired form of TTP is thought to be due to an autoantibody against ADAMTS13, and the inherited form is due to a genetic deficiency of ADAMTS13. The hemolytic uremic syndrome is thrombotic microangiopathy associated with severe renal impairment and normal or slightly reduced ADAMTS13 activity. The most common form (typical HUS) is associated with bloody diarrhea due to Shiga toxin-producing *Escherichia coli. *Atypical HUS is less common. It is caused by a dysregulation of the alternative complement pathway, which leads to increased complement activity.

Thrombotic microangiopathy is a clinicopathologic diagnosis. The constellation of thrombocytopenia, anemia and red blood cell fragmentation (i.e., schistocytes) on the blood film is sufficient to make the diagnosis. The finding of concomitant anemia and thrombocytopenia should prompt a request for a peripheral blood film to look for red blood cell fragmentation. For TTP, the full pentad of signs and symptoms (thrombocytopenia, schistocytic anemia, neurologic impairment, renal impairment, and fever) is present in only 5% of patients [18]. The first two criteria i.e., thrombocytopenia and schistocytic anemia, are enough to initiate treatment. Other laboratory abnormalities in patients with thrombotic microangiopathy are reticulocytosis, elevated lactate dehydrogenase, unconjugated hyperbilirubinemia, and low haptoglobin. The direct antiglobulin test is usually negative, and coagulation tests (international normalized ratio (INR) and partial thromboplastin time) that appear normal may become elevated in patients with very severe diseases associated with disseminated intravascular coagulation. 

Once thrombotic microangiopathy is suspected, a thorough search for secondary causes is important to help guide treatment. This should include a pregnancy test for females of childbearing potential and careful questioning about drug exposures, HIV risk factors, and other infections. The ADAMTS13 testing should be done before the institution of plasma therapy to avoid false-negative results. It should also be noted that some of the tests required in the differential diagnosis (e.g., ADAMTS13 activity assay) are not available at all institutions. If rapid ADAMTS13 testing is not possible, the platelet count; combined hemoLysis variable; absence of active cancer; absence of stem-cell or solid-organ transplant; mean corpuscular volume (MCV); INR; creatinine (PLASMIC) score, a seven-component prediction tool that can accurately and reliably predict the probability of severe ADAMTS13 deficiency, can be used [19].

Thrombotic thrombocytopenic purpura is a hematologic emergency. Untreated, it is associated with mortality as high as 90%; with plasma exchange, mortality can be reduced to 20%. Once TTP is suspected, plasma exchange should be instituted promptly. Corticosteroids are frequently added to plasma exchange as part of initial therapy [18]. The typical regimen is prednisone 1 mg/kg orally followed by a rapid taper over three to four weeks once a normal platelet count has been achieved. Higher doses may be used for patients with more severe diseases. For patients with atypical HUS, plasma exchange should be administered initially because the clinical features are often indistinguishable from TTP. The diagnosis of atypical HUS should be considered in patients with substantial renal impairment who do not respond to plasma exchange and who do not have severe ADAMTS13 deficiency. For those patients, eculizumab, a monoclonal antibody against the terminal complement component C5, should be considered [17]. Evidence from a retrospective study suggests that relapsed or refractory TTP can be managed successfully by increasing the intensity or frequency of plasma exchanges and adding or increasing doses of immunosuppressant therapy, including corticosteroids. Rituximab has been shown to be effective in refractory TTP, and some investigators have used it as initial therapy. Other treatments include cyclosporine, cyclophosphamide, vincristine, N-acetylcysteine, and splenectomy, which can reduce the rate of relapse in patients with recurrent TTP. Emerging therapies include caplacizumab, a novel anti-von Willebrand factor nanobody, and recombinant ADAMTS13 [18].

Disseminated intravascular coagulation (DIC) 

**Figure 2:**
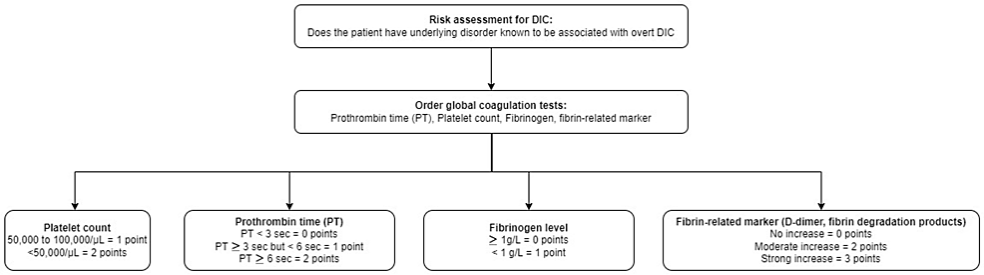
DIC diagnosis score ≥ 5 points: compatible with overt DIC, repeat scoring daily < 5 points: suggestive of non-overt DIC, repeat scoring within the next one to two days DIC: Disseminated intravascular coagulation

Disseminated intravascular coagulation is a clinicopathological diagnosis of a disorder that is defined by the International Society on Thrombosis and Hemostasis (ISTH) as “an acquired syndrome characterized by the intravascular activation of coagulation with loss of localization arising from different causes” [3]. This condition typically originates in the microvasculature and can cause damage of such severity that it leads to organ dysfunction. The most common causes of DIC are severe sepsis, usually with septic shock; disseminated malignancy, most classically with mucin-secreting pancreatic adenocarcinoma; pregnancy with various severe complications, including sepsis, placental abruption, and eclampsia; cases of life-threatening illnesses characterized by systemic inflammatory response syndrome (SIRS) with hypotension and multiorgan dysfunction. The consumption of the coagulation proteins and platelets produces a bleeding tendency with thrombocytopenia, a prolonged prothrombin time (PT), a partial thromboplastin time (aPTT), hypofibrinogenemia, and elevated levels of fibrin degradation products that can be detected with a D-dimer test and a risk assessment for DIC to identify patients with overt and non-overt DIC (Figure 2) [3]. The physiologic anticoagulants are also consumed in the process of inhibiting the many activated coagulation factors. In fulminant DIC, the consumption and diminished supply of platelets and coagulation proteins usually result in oozing at vascular access sites and wounds but occasionally cause profuse hemorrhage. A diagnostic scoring system for DIC has been developed which aids in diagnosing high-risk cases for developing DIC. The cornerstone for managing DIC remains the management of the underlying cause (e.g., sepsis), replacement of coagulation proteins and platelets in patients who are bleeding, platelet transfusion to maintain a platelet level of > 50,000, transfusion of fresh frozen plasma (FFP) to maintain aPTT < than 1.5 times the normal, and fibrinogen to maintain > 1.5 g per liter. The use of antifibrinolytic agents is contraindicated in the management of disseminated intravascular coagulation since the fibrinolytic system is required in recovery to ensure the dissolution of the widespread fibrin [3].

Trauma, bleeding, and postoperative thrombocytopenia

Thrombocytopenia is a frequent finding in the ICU, particularly among adults. The Prostate Testing for Cancer and Treatment (PROTECT) trial study used platelet count definitions of <150 x 109/L for mild thrombocytopenia, <100 x 109/L for moderate thrombocytopenia, and <50 x 109/L for severe thrombocytopenia; they found incidence rates of 15.3%, 5.1%, and 1.6%, respectively, for mild/moderate/severe incidence rates in medical-surgical ICUs [4].

Although thrombocytopenia has many potential causes, the risk factors most commonly observed with ICU-acquired thrombocytopenia are illness severity, sepsis, renal failure, vasopressor medications, and organ dysfunction [20]. Hemorrhages are responsible for 30% to 40% of all trauma-related mortality, with 60% of hemorrhage deaths occurring within three hours of hospital admission and 94% of hemorrhage deaths occurring within 24 hours [20]. Therefore, in order to avoid preventable trauma-related deaths, patients must be subject to appropriate initial fluid resuscitation treatments. Fluid resuscitation must be designed to correct thrombocytopenia and other coagulopathies, but the frequency of complications in ICU thrombocytopenia makes establishing discrete, optimal resuscitation strategies difficult [21]. Conventional damage control resuscitation (DCR) in patients with massive hemorrhage/severe trauma usually begins with a rapid infusion of 1L to 2L of crystalloid fluids, with subsequent transfusion of compatible red blood cells (RBC). The latter transfusion does not contain FFP or platelets in conventional DCR [20]. This technique, however, was found to frequently lead to various adverse outcomes such as abdominal compartment syndrome, acute respiratory distress syndrome, multiple organ failure, and dilutional coagulopathy. A renewed DCR strategy has been recently introduced to include simultaneous transfusion of FFP and platelets along with the initial RBC units while minimizing crystalloid infusion. This aggressive new strategy is essential to combat and correct thrombocytopenia or other coagulopathies, and also has the potential to ensure good outcomes for trauma patients. In the updated DCR, rapid hemorrhage control treatment advises an early administration of a balanced 1:1:1 ratio of FFP, platelets, and RBCs [20].

Postoperative thrombocytopenia is a common complication that occurs after surgery. The postoperative period includes numerous treatments and interventions, including blood transfusions and medication use (heparin in particular). Thrombocytopenia within four days following surgery is fairly common and caused by hemodilution and increased platelet consumption prior to thrombopoietin-induced platelet recovery [22]. Under standard conditions, thrombopoietin physiology induces the platelet count rebound and potentially an overshoot. Thrombocytopenia occurrence after postoperative day five includes a much broader list of causes, which can be broken into consumptive and destructive disorders. Some consumptive disorders include infection-associated disseminated intravascular coagulation and postoperative thrombotic thrombocytopenic purpura, while destructive disorders include drug-induced immune thrombocytopenia and post-transfusion purpura [22]. Understanding the timing and severity of postoperative thrombocytopenia can provide a practical approach to a frequently challenging consultation.

Understanding the dynamics of platelet count levels and their temporal relevance is crucial, as early postoperative platelet loss is informative about the magnitude of blood loss or trauma. Subsequently, a slow and gradual fall is indicative of consumptive coagulopathy or bone marrow failure, and an abrupt decrease in platelet count at least one-week post-operation strongly suggests immunological causes such as heparin-induced thrombocytopenia [2].

Transfusion thresholds

The severity of the condition is important when determining the criteria for transfusion prior to certain medical procedures. According to the American Association of Blood Banks (AABB), the guidelines for platelet transfusions are as follows (Table 6) [23]: platelet count of less than 50x109/L is an indication for transfusion prior to most surgeries excluding neurosurgery, elective diagnostic lumbar puncture, severe bleeding requiring massive transfusions. Additionally, fewer than 20 x109/L is the threshold used before a central vein catheter or urgent lumbar punctures can be performed [4]. Prophylactic transfusion is indicated when platelet levels become fewer than 10 x109/L. It is important to understand the various thresholds because many patients admitted to the ICU may develop thrombocytopenia or are admitted with the condition. The AABB cannot recommend for or against platelet transfusion for patients receiving antiplatelet therapy who have intracranial hemorrhage (traumatic or spontaneous). There are no specific recommendations for platelet transfusion for bone marrow biopsy and paracentesis [4].

**Table 6:** Platelet transfusion threshold for various procedures MTP: Massive transfusion protocol, TBI: Traumatic brain injury, ICH: Intracranial hemorrhage, IVD: Intraventricular drain

Severity of thrombocytopenia	Platelet threshold for transfusion	Procedures
Severe <50 x10^9^/L	10 x10^9^/L	Prophylaxis in adults
Severe <50 x10^9^/L	20 x10^9^/L	Prior to bronchoscopy and lavage, prior to elective central venous catheter, prior to urgent diagnostic lumbar puncture
Moderate 50-100 x10^9^/L	50 x10^9^/L	Severe bleeding, MTP prior to chest tube insertion for thoracentesis, prior to elective diagnostic lumbar puncture, prior to major elective surgery excluding neurosurgery
Mild 100-150 x10^9^/L	100 x10^9^/L	Prior to neurosurgery,TBI, ICH, prior to IVD insertion

With the advancements in image-guided techniques such as bedside ultrasonography for central line placements, thoracentesis, paracentesis, and procedures by interventional radiology, there has been an increasing trend toward a restrictive approach for platelet and plasma transfusions in preparation for invasive bedside procedures [24]. According to Society for Interventional Radiology guidelines (SIR), procedures are classified as low risk and high risk (Table 7). Multiple randomized control trials (RCT) have shown that routine preprocedural testing did not help identify patients who are at high risk for bleeding and the risk of hemorrhagic complications requiring transfusions or termination of procedure was found to be low. In addition, pre-procedure testing was found to delay the commencement of the procedure and burdened the laboratories. Hence SIR does not recommend pre-procedural coagulation testing for low-risk procedures. For high-risk procedures, recommendations to date still follow i.e., correcting coagulopathy by transfusion of platelet and plasma to maintain a platelet count of 50,000μ/L and plasma for INR <1.5 -1.8. It is highly implausible to have large RCTs to study the risks and benefits of liberal vs restrictive transfusion strategies without exposing the patients to unnecessary risk of transfusion-related complications and hence these queries still remain unanswered. 

**Table 7:** Risk stratification for interventional radiology procedures as per Society for Interventional Radiology guidelines IVC: Inferior vena cava, PICC: Peripherally inserted central catheter, CNS: Central nervous system, DVT: Deep vein thrombosis, PE: Pulmonary embolism

Risk assessment	Procedures
Low-risk procedures	Catheter exchanges (gastrostomy, biliary, nephrostomy, abscess); diagnostic arteriography and arterial interventions: peripheral, sheath <6 French, embolotherapy; diagnostic venography and select venous interventions: pelvis and extremities; dialysis access interventions; facet joint injections and medial branch nerve blocks (thoracic and lumbar spine); IVC filter placement and removal; lumbar puncture; non-tunneled chest tube placement for pleural effusion; non-tunneled venous access and removal (including PICC placement); paracentesis and thoracentesis; peripheral nerve blocks, joint, and musculoskeletal injections; sacroiliac joint injection and sacral lateral branch blocks; superficial abscess drainage or biopsy (palpable lesion, lymph node, soft tissue, breast, thyroid, superficial bone, extremities and bone marrow); transjugular liver biopsy; trigger point injections including piriformis; tunneled drainage catheter placement; tunneled venous catheter placement/removal (including ports)
High-risk procedures	Ablations: solid organs, bone, soft tissue, lung; arterial interventions: >7 French sheath, aortic, pelvic, mesenteric, CNS; biliary interventions (including cholecystostomy tube placement); catheter-directed thrombolysis (DVT, PE, portal vein); deep abscess drainage (lung parenchyma, abdominal, pelvic, retroperitoneal); deep non-organ biopsies (spine, soft tissue in intraabdominal, retroperitoneal, pelvic compartments); gastrostomy/gastrojejunostomy placement IVC filter removal complex; portal vein interventions; solid organ biopsies; spine procedures with risk of spinal or epidural hematoma (kyphoplasty, vertebroplasty, epidural injections, facet blocks cervical spine); transjugular intrahepatic portosystemic shunt; urinary tract interventions (including nephrostomy tube placement, ureteral dilation, stone removal); venous interventions: intrathoracic and CNS interventions

Alternatives and additions to platelet transfusions

Antifibrinolytic Therapy: Tranexamic Acid 

The antifibrinolytic agent, tranexamic acid (TXA), has been found to reduce bleeding risk and transfusion needs during surgery, mortality risk in trauma patients, and found to be beneficial in patients with thrombocytopenia with MDS and mucous membrane bleeding (use with caution in ischemic heart disease and hematuria patients) as recommended by various existing guidelines such as National Institute for Health and Clinical Excellence (NICE), Cochrane review (Wardrop et al., 2013) and British Committee for Standards in Hematology (BCSH) guidelines [25]. The Clinical Randomisation of an Antifibrinolytic in Significant Haemorrhage 2 (CRASH-2) trial, 2013 showed that if given within one hour, TXA reduces mortality in bleeding trauma patients. Specifically, tranexamic acid reduces the risk of bleeding to death by about one-third, with no increase in side effects. Use of TXA over three hours of injury is ineffective. The CRASH-3 trial in 2019 was conducted to study the effect of TXA on traumatic brain injury (TBI) patients with mild-moderate head injury (Glasgow coma score (GCS) 13-15) and evidence of intracranial bleeding on initial CT head. Use of TXA within three hours of the injury was found to reduce mortality secondary to head injuries by reducing intracranial bleeding [26]. The Haemorrhage Alleviation With Tranexamic Acid- Intestinal System (HALT-IT) trial, 2020 concluded that tranexamic acid was not recommended to be used in gastrointestinal bleeding and had increased venous thromboembolic complications such as DVT and PE [27]. 

Desmopressin

Desmopressin is a synthetic analog of vasopressin that stimulates factor VIII release from endothelial stores and increases von Willebrand factor activity thus promoting coagulation. The use of desmopressin has been studied in trauma patients receiving aspirin, perioperative bleeding, patients with uremia, and von Willebrand disease [25].

Thrombopoietin Receptor Agonists and Other Therapies

Romiplostim and eltrombopag are approved to be used in patients with ITP. Their use in MDS/non-Hodgkin lymphoma, and severe aplastic anemia is controversial due to increases in blast counts and clonal progression and warrants more studies. In patients with chronic liver disease, the use of eltrombopag is found to be associated with portal vein thrombosis. Erythropoietin in hematopoietic stem cell transplant (HSCT) patients has been shown to reduce the need for red cell and platelet transfusions [25]. 

Thromboelastography (TEG)

Thromboelastography (TEG) is a non-invasive, in vitro diagnostic study that quantitatively measures the clotting ability of the blood. It quantifies the real-time changes of the viscoelastic properties of the blood during the process of clotting under low sheer stress in the form of graphical representation [28]. Normally citrated blood samples at room temperature are used for TEG study. Various modifications of the classic TEG study exist to improve its diagnostic value and usage. When whole, non-citrated blood samples are used it is called native blood TEG or non-activated thromboelastometry (NATEM) but it should be tested immediately. Rapid TEG (r-TEG) utilizes tissue factor to activate blood coagulation. This is faster than the conventional TEG where kaolin cephalin reagent is used for blood coagulation. Rapid TEG is faster and is used to manage massive transfusions in trauma patients. The TEG platelet mapping assesses platelet aggregation in the presence of adenosine diphosphate or arachidonic acid and is used to predict the antiplatelet activity of agents such as aspirin and clopidogrel. The TEG with added heparinase (hTEG) measures the effects of heparin on blood coagulation and helps manage heparin reversal as needed. Rotational thromboelastometry (RoTEM) is a variation of TEG which incorporates an oscillating pin while the cup remains in a stable position. 

**Figure 3:**
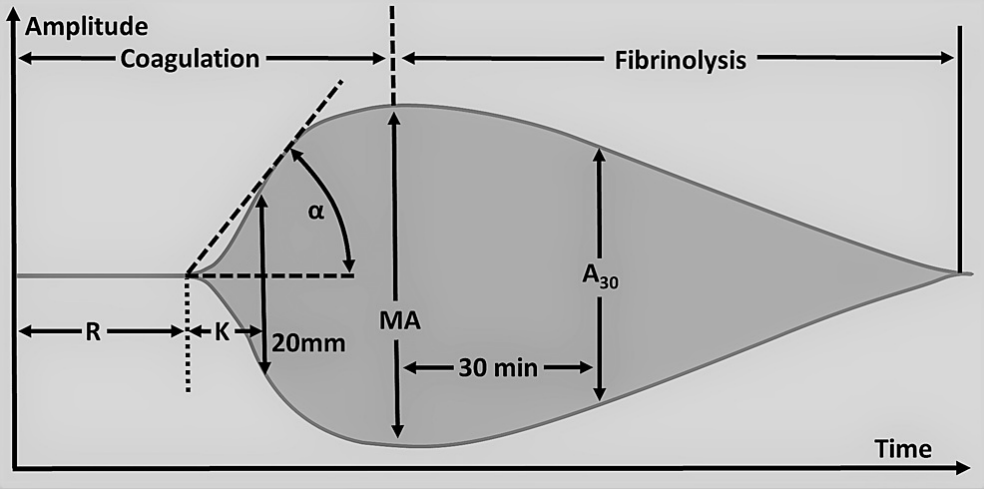
Thromboelastography parameters. R: Reaction time, K: K-time/Kinetics, A/α: Alpha angle, MA: Maximum amplitude, A_30: _Amplitude at 30 minutes after the beginning of the test Contributed by Mikael Häggström, Creative Commons Attribution 4.0 International License.

Qualitative analysis of the TEG tracing is obtained during the test, and qualitative analysis of TEG includes the measurement of five parameters, namely reaction time (R), kinetics (K), alpha angle (A), maximum amplitude (MA), and lysis at 30min (LY30) (Table 8). Abnormalities in these parameters indicate suboptimal hemostasis and coagulation. Reaction time (R ) is the time taken to the first detectable clot formation and the prolongation of time indicates the deficiency of coagulation factors (Figure 3). This can be treated with FFP, prothrombin complex concentrate ( PCC), and reversal of anticoagulation that the patient might be taking that is causing bleeding complications. Kinetics (K) is the time from the first clot formation to the formation of a clot with a certain level of strength. The alpha angle (A) is the angle between R and the imaginary line from the time of clot initiation to the point of maximum clot formation speed. Prolongation of the K time or its decrease and the alpha angle suggests a deficiency of fibrinogen and is managed by transfusion of cryoprecipitate or fibrinogen. Maximum amplitude (MA) is the maximum amplitude of the TEG curve. Low MA is secondary to qualitative platelet defects or quantitative deficiency of platelets and is corrected by desmopressin or transfusion of platelets. Lysis at 30min (LY30) is the percentage of the amplitude reduction 30 minutes after reaching the maximum amplitude. An increased LY value indicates activated fibrinolysis and is treated by agents such as aminocaproic acid or tranexamic acid (TXA). Rotational thromboelastometry (RoTEM) assay uses different terminology to define the TEG parameters: clotting time (CT) instead of R, clot formation time (CFT) for K, maximum clot firmness (MCF) for MA, and clot lysis (CL) for LY. 

**Table 8:** Thromboelastography (TEG) parameters with normal values Contributed by Maxim Shaydakov, Creative Commons Attribution 4.0 International License.

Parameter	Description	Reference value	Biological meaning
Reaction Time (R)	Time from the beginning of the test to the first detectable clot formation (amplitude of 2mm)	5-10 minutes	Activation phase: Time that is needed to activate the intrinsic pathway and initiate fibrin deposition. Depends on the concentration and function of the coagulation factors, reflects the ability of the blood to generate thrombin. Maybe affected by congenital or acquired coagulation factors deficiency and anticoagulation therapy.
Kinetics (K)	Time from the beginning of clotting to the formation of the clot with a certain level of strength corresponding to the amplitude of 20 mm	1-3 minutes	Amplification phase: The speed of initial fibrin deposition and cross-linking. Depends on the concentration of fibrinogen and its activation (the abundance of thrombin). To a lesser extent dependent on platelets.
Alpha Angle (A)	Angle between R and imaginary line from the time of clotting initiation to the point of the maximal clot formation speed. Closely related to K time.	53-72 degrees	Propagation phase: Characterizes the maximum speed of thrombin generation, fibrin deposition, and cross-linking (clot growth and strengthening). Depends on the concentration of fibrinogen and to a lesser extent on platelets.
Maximum Amplitude (MA)	Maximal amplitude of the TEG curve	50-70 mm	Termination phase: The maximal mechanical strength of the clot. Depends on the platelet abundance, GPIIb/IIIa interactions, fibrin cross-linking, and clot contraction. May be affected by thrombocytopenia, thrombocytopathy, and antiplatelet agents.
Lysis at 30 minutes (A30 or LY30)	Percentage of the amplitude reduction 30 minutes after reaching maximal amplitude	0-8%	Fibrinolysis phase: The speed of endogenous fibrinolysis. Depends on the presence of the plasmin, plasminogen, and its activators in the blood samples.

Various reagents are used to assess different functionality of clotting as described in Table 9 below referring to various RoTEM subtypes such as intrinsically-activated test using ellagic acid (INTEM), extrinsically-activated test with tissue factor (EXTEM), heparinase-modified thromboelastometry (HEPTEM), a modified EXTEM assay incorporating aprotinin to stabilize the clot against hyperfibrinolysis (APTEM), fibrin-based extrinsically activated test with tissue factor and the platelet inhibitor cytochalasin D (FIBTEM), and NATEM. The INTEM activates the intrinsic coagulation pathway; EXTEM activates the extrinsic coagulation pathway. The HEPTEM determines the effect of heparin on bleeding and guides heparin reversal using protamine; APTEM predicts the effect of hyperfibrinolysis and guides the use of TXA; FIBTEM differentiates between hypofibrinogenemia and platelet deficiency. 

**Table 9:** RoTEM Subtypes Contributed by Maxim Shaydakov, Creative Commons Attribution 4.0 International License. RoTEM: Rotational thromboelastometry, INTEM: Intrinsically-activated test using ellagic acid, EXTEM: Extrinsically-activated test with tissue factor, HEPTEM: Heparinase-modified thromboelastometry, APTEM: a modified EXTEM assay incorporating aprotinin to stabilize the clot against hyperfibrinolysis, FIBTEM: Fibrin-based extrinsically activated test with tissue factor and the platelet inhibitor cytochalasin D, NATEM: Non-activated thromboelastometry

Test	Description
INTEM	Reagents: Phospholipids and ellagic acid. Ellagic Acid is a natural phenol that can activate Factor XII (intrinsic coagulation pathway).
EXTEM	Reagent: Tissue factor. Activates extrinsic coagulation pathway.
HEPTEM	Reagents: Phospholipids, ellagic acid, heparinase. Heparinase is an enzyme that neutralizes heparin. Serves as an adjunct to INTEM to determine the impact of heparin on coagulopathy to guide protamine sulphate therapy.
APTEM	Reagents: Tissue factor, aprotinin. Aprotinin is a bovine pancreatic trypsin inhibitor that also inhibits plasmin. Serves as an adjunct to EXTEM to predict the clinical effect of fibrinolysis inhibitor in case of hyperfibrinolysis. Mimics treatment with tranexamic acid.
FIBTEM	Reagent: Cytochalasin D. Cytochalasin D is a fungus-produced alkaloid that is able to inhibit platelet activity. Helps to differentiate between hypofibrinogenemia and platelet deficiency.
NATEM	Reagent: None. Whole blood with no additional reagents.

The TEG has been widely implemented into clinical practice for a comprehensive assessment of coagulopathy including coagulation cascade, platelet function, and fibrinolysis. It is a quick real-time bedside point of care test that can be readily used in trauma patients to assess the need for appropriate blood product transfusions and reversing coagulopathy. It is widely used to assess transfusion needs and detect dynamic changes in coagulation during resuscitation such as in cardiac surgery, postoperative, and liver transplant patients. In surgical patients, the use of TEG platelet mapping can detect platelet inhibition by antiplatelet agents such as aspirin and Plavix. Its use in extracorporeal membrane oxygenation (ECMO) patients is being studied to better guide anticoagulation over aPTT and has been shown to reduce heparin doses in a small RCT [28]. 

Thrombocytopenia in pregnancy

Thrombocytopenia in pregnancy is the second most common hematological abnormality after anemia. The prevalence of thrombocytopenia at the end of pregnancy is between 6.6% and 11.6%. Knowledge of its various causes and appropriate management is key for a safe pregnancy and healthy outcomes. The varied causes of thrombocytopenia in pregnancy are listed in Table 10 [29]. Incidental thrombocytopenia of pregnancy also referred to as gestational thrombocytopenia accounts for up to 70% to 80% of cases and is most common in the second to the late third trimester of pregnancy. These patients usually have low to moderate thrombocytopenia. Severe thrombocytopenia warrants further investigation to rule out other causes of thrombocytopenia. Gestational thrombocytopenia usually resolves one to two months after delivery. Immune thrombocytopenic purpura is the second most common cause of thrombocytopenia in pregnancy. Platelet counts of less than 100 x109/L early in pregnancy and declining as gestation progresses is typical of ITP and patients might report a history of ITP. Various treatment modalities are recommended if platelet counts are less than 30x109/L or the patient is symptomatic with bleeding, namely prednisone, intravenous immunoglobulin IvIg, a combination of steroids plus IvIg, azathioprine for refractory cases to first-line therapies, and splenectomy to induce remission in the second trimester for patients refractory to above treatments. Most other agents used in non-pregnant ITP patients are not used in pregnancy due to toxicities. Platelet counts of at least 80-100 x 109/L are recommended for epidural anesthesia and 50 x 109/L for cesarean section. 

**Table 10:** Causes for pregnancy-related thrombocytopenia HELLP syndrome: Hemolysis, elevated liver enzymes, low platelet count syndrome, ITP: Immune thrombocytopenic purpura, VWD: von Willebrand disease, TTP: Thrombocytopenic thrombotic purpura, HUS: Hemolytic uremic syndrome, SLE: Systemic lupus erythematosus

Pregnancy-related thrombocytopenia	Etiology
Pregnancy-specific Isolated thrombocytopenia	Gestational thrombocytopenia
Pregnancy-specific thrombocytopenia associated with systemic disorders	Preeclampsia HELLP syndrome, acute fatty liver of pregnancy
Not pregnancy-specific Isolated thrombocytopenia	Primary immune thrombocytopenia–ITP, secondary ITP, drug-induced thrombocytopenia, type IIb VWD congenital
Not pregnancy-specific thrombocytopenia associated with systemic disorders	TTP/HUS, SLE, antiphospholipid antibody syndrome, viral infections, bone marrow disorders, nutritional deficiency, splenic sequestration (liver diseases, portal vein thrombosis, storage disease, etc.

Thrombotic microangiopathies of pregnancy including preeclampsia; hemolysis, elevated liver enzymes and low platelets (HELLP) syndrome; and acute fatty liver of pregnancy (AFLP) have clinical and laboratory features that overlap with thrombotic microangiopathies that are not pregnancy specific such as TTP/ HUS (Table 11). These are hematological emergencies and are managed in critical care units. Once a diagnosis of preeclampsia is established, patients are managed with magnesium infusions, monitoring for seizures, blood pressure control, and close monitoring of coagulopathy. The HELLP syndrome is a life-threatening variant of severe pre-eclampsia. Intravenous dexamethasone has been used for the treatment of severe HELLP with platelet counts less than 50x109/L. The AFLP is a life-threatening complication of the third trimester of pregnancy which is characterized by laboratory abnormalities of hemolysis, thrombocytopenia, elevated liver enzymes, hypoglycemia, elevated ammonia, and reduced fibrinogen. Treatment is supportive and includes maternal stabilization and correct coagulopathy. The definitive treatment of preeclampsia with severe features, eclampsia, and HELLP syndrome is delivery of the fetus [10]. 

**Table 11:** Thrombotic microangiopathies in pregnancy HTN: Hypertension, AST: Aspartate aminotransferase, LDH: Leucocyte dehydrogenase

Thrombotic microangiopathies	Clinical and lab findings	Management
Preeclampsia	HTN, proteinuria	IV magnesium, delivery of the fetus depending on gestational age, +/- corticosteroids for fetal lung maturity
HELLP syndrome	HTN, proteinuria, ↑AST, ↑bilirubin, ↑ LDH, normal glucose, thrombocytopenia	
AFLP	+/-HTN, +/- proteinuria, +/-thrombocytopenia, ↑ammonia, ↓ glucose, ↓ fibrinogen, ↓antithrombin, ↑PT/INR, ↑AST, ↑bilirubin, ↑ LDH	Maternal stabilization, correct coagulopathy, delivery
TTP/ HUS	Fever, thrombocytopenia, hemolytic anemia, neurological abnormalities, renal dysfunction, +/- proteinuria, +/- HTN	Daily plasma exchange until platelet normalizes, +/- IV or PO steroids

Future directions

The use of fibrinolytics has been extensively studied and used in various ICU scenarios for TBI and trauma as shown in CASH-2 and CASH-3 trials and found to have improved outcomes in patients with mild to moderate TBI by a third. However, the HALT-IT trial has found that TXA can lead to DVT and PE in patients with gastrointestinal (GI) bleed. With such varied outcomes in the use of TXA, it is highly recommended that the safety and efficacy of TXA be extensively researched before using them in various clinical scenarios. 

Given the complex pathophysiology in sepsis-related coagulopathy, one has to maintain a tenuous balance between bleeding and a high risk of thrombosis in these patient populations. Assessing the appropriate timing of antiplatelet agents in these patients is another area of ongoing research. 

The use of TEG is widely utilized in various clinical scenarios such as trauma, intraoperative and postoperative patients to guide appropriate product transfusions and titration of heparin dosing in ECMO patients. Using a novel TEG-based scoring system was suggested to diagnose DIC and help assess the appropriate correction of coagulopathy. The TEG study to diagnose intrinsic coagulopathy by heparin and low molecular weight heparin (LMWH) that can help in patients with intracranial bleeding by emergently reversing heparin activity has been under investigation. Patients with ST-elevation myocardial infarction (STEMI) and of percutaneous coronary intervention (PCI), are treated with standard dosing of antiplatelet agents all across the board, and resistance to antiplatelet agents is not usually considered during initiation and management of these patients. More studies are necessary to implement TEG to measure the effect of antiplatelet therapy, diagnose resistance, and predict the bleeding and thromboembolic risk in these cardiac patients. The TEG parameters may be used for risk stratification of critically ill, trauma, and oncologic patients who are at increased risk of developing venous thromboembolism (VTE). 

## Conclusions

This paper presents a concise and comprehensive review of the various etiologies, pathophysiology, and management of thrombocytopenia in specific ICU settings. Coagulopathy in ICU is a very challenging disorder with complexities in reversing the onboard agents causing coagulopathy, identifying the underlying etiology and specific presentation of the patient. There are a plethora of issues associated with transfusion of blood products and recent research is growing towards a conservative over the liberal approach to transfusions. This growing evidence of the conservative blood product transfusion approach is associated with improved morbidity by reducing various transfusion-related complications such as infections, febrile reactions, transfusion-associated circulatory overload (TACO), and transfusion-related acute lung injury (TRALI) as well as an economic transfusion of essential blood products. 

To this day, questions remain unanswered about the specific threshold for transfusion of blood products appropriately. These thresholds vary according to the severity and complexity at the time of presentation. Large RCTs are required to unequivocally assess the platelet and blood product transfusion thresholds. The results of these research activities will hopefully lead to improved guidelines for platelet transfusion thresholds and treatment modalities to help clinicians diagnose and manage effectively. The findings will further assist interventional radiologists and intensivists perform urgent/emergent procedures safely while using image guidance to prevent unnecessary transfusions of blood products and longer wait times to get the lab results, thereby allowing providers to act appropriately.
